# Comparison of diagnostic values of transvaginal sonography with laparoscopic and histological results in the evaluation of uterosacral ligaments’ involvement in endometriosis patients

**DOI:** 10.22088/cjim.13.4.705

**Published:** 2022

**Authors:** Pegah Kamkarfar, Roya Shahriyaripoor, Samaneh Rokhgireh, Seyed Reza Saadat Mostafavi, Shahla Chaichian, Abolfazl Mehdizadeh Kashi

**Affiliations:** 1 Department of Obstetrics and Gynecology, Iran University of Medical Sciences, Tehran, Iran; 2 Endometriosis Research Center, Iran University of Medical Sciences (IUMS), Tehran, Iran; 3 Department of Radiology, Iran University of Medical Sciences, Tehran, Iran

**Keywords:** Endometriosis, Uterosacral ligament, Transvaginal sonography (TVS), Laparoscopy

## Abstract

**Background:**

Endometriosis is one of the most common gynecological disorders, which causes pain and reduces fertility. An accurate diagnostic technique would be helpful in the management of these patients preoperatively. The objective of this study was to do a comparative evaluation of uterosacral involvement in deep infiltrative endometriosis by transvaginal sonography (TVS) and laparoscopic biopsy.

**Methods:**

TVS and laparoscopy were done in all patients suspected to have endometriosis. TVS examination was carried out to identify endometriotic lesions, and in suspicious laparoscopic views, biopsy was done and laparoscopic findings were confirmed by pathologic report. Then, TVS and pathological findings in laparoscopy were compared and data analyzed by SPSS Version 23.

**Results:**

In our study on 80 patients, the mean age was 34.47 ± 5.94 (mean ± SD) years. Comparison of ultrasound with laparoscopic examinations showed that ultrasound as the gold standard method, has sensitivity, specificity, and positive and negative predictive values of 93%, 65%, and 87%, and 78.9%, respectively, while in the diagnosis of increased uterosacral ligament thickness showed 82%, 100%, and 100% and 6.66%, respectively. While in the diagnosis of nodules in the uterosacral ligament, 100% for all four parameters in the diagnosis of endometrioma in the ovaries, and 71%, 96.4%, and 97.3% and 64.2%, respectively, in the diagnosis of rectal, bladder, and ureteral involvement.

**Conclusion:**

TVS can be used in the diagnosis of endometriosis by examining the increase in the thickness of the uterosacral ligament and the presence of hypoechoic nodules in it; also, this method demonstrates acceptable sensitivity and specificity in ovarian endometrioma.

Endometriosis is one of the most common gynecologic reproductive disorder, in which the endometrial tissue is aberrantly located outside the uterus (1). According to the Endometriosis Society consensus, the endometriosis lesions penetrate deeper than 5mm into the peritoneum, bladder, vagina and other areas of the pelvis (2). With regard to the anatomical site of implantation, it involves symptoms such as acyclic pelvic pain, deep dyspareunia, dysmenorrhea and infertility (3, 4). Pelvic endometriosis can be classified into three categories: superficial, ovarian, and deep peritoneal infiltration (5). The prevalence of endometriosis has been reported in 15%–30% of patients (6, 7). The most common areas of involvement are the uterosacral ligaments (USLs) (8-10). USL involvement could lead to many clinical symptoms, including chronic pelvic pain and dyspareunia (11).

Generally, endometriosis may clinically occur with one or more symptoms, including chronic pelvic pain, dysmenorrhea, dyspareunia, or infertility, depending on the location of the lesions. When the bowel and bladder are affected, patients may experience pain at the time of urination or defecation (12). Prevalence of endometriosis in women with pelvic pain has been estimated at 5%–20%, in infertile women 20%–40%, and 5%–15% in premenopausal women. In white women aged 25–40 years, the prevalence of endometriosis varies among populations, while in the reproductive age, it has been found to be about 10% (13-15). Different studies have evaluated that there is an approximate of 7 years between the emergence of the first symptoms (in the ovaries, uterus, fallopian tubes and round ligaments) and clinically confirmed diagnosis of endometriosis (16). Other areas with less prevalence include vagina, cervix, and rectovaginal septum, usually caused by infiltration (17). The appearance and size of lesions during surgery vary (18, 19). Evidence suggests the influence of family history of endometriosis among women with the disease (20). Surgery is a gold standard method for endometriosis. Ultrasound is used as a diagnostic imaging tool in women suspected to have endometriosis. This method has been suggested for the diagnosis of deep endometriosis due to its high resolution, low cost, and relatively low discomfort. Based on studies conducted so far, transvaginal-transrectal ultrasound is an effective technique in the accurate diagnosis of endometriosis with USL involvement. Color Doppler was also used to evaluate ovarian endometrium vascularity (21). Laparoscopy is one of the most important diagnostic tools in women for evaluating fallopian tubes and endometriosis, and other abdominal disorders and plays an important role in final decision making for the initiation of infertility treatment (18). As mentioned above, several studies have evaluated these methods separately in endometriosis patients. However, there is not any report about comparison of the sensitivity and specificity of them so far. Consequently, in this study, for the first time in Iran, we intended to evaluate and compare the sensitivity and specificity of transvaginal sonography with laparoscopic and histological results in the evaluation of uterosacral ligaments’ involvement in endometriosis patients.

## Methods

**Study setting: **This is a cross-sectional study performed on patients with suspected clinical symptoms of endometriosis (chronic pelvic pain, dysmenorrhea, or dyspareunia) at Rasoul Akram Hospital in Tehran during 2019–2020. It was approved by the Ethics Committee of Iran University of Medical Sciences (IR.IUMS.FMD.REC1398.383). A total of 80 patients were included in this study. Inclusion criteria included all patients with high clinical suspicion of endometriosis and aged 18–49 years, and exclusion criteria included virginity or any conditions in which the patient cannot undergo transvaginal ultrasound or laparoscopic surgery. 

Patients were evaluated for dysmenorrhea, pelvic pain, and dyspareunia in terms of intensity (scoring from 0 to 10 based on the visual analog scale) and duration (months or years) of pain. All patients were confirmed by their physicians before participating in the study. Also, all patients' clinical information was collected from their files based on the relevant checklist that had been prepared in advance. Sampling method in this study is random sampling and the sample size was calculated based on the formula below 80 people.

$n=\frac{{\left(Z1-\frac{\alpha }{2}+Z1-\beta \right)}^{2}(\alpha_{1}^{2}+\alpha_{2}^{2})}{d^{2}}$

(In this formula, the probability of the first type error is 0.05 and the probability of the second type error is 0.2(.

**Transvaginal sonography evaluation: **An expert radiologist in endometriosis diagnosis, examined the patients with vaginal ultrasound in OB presentation with a GE VOLOSON sonography device with a transvaginal probe. Ultrasound findings include nodular aggregations or the presence of irregular hypoechoic nodules, thickening of the wall, or retractable masses and hypoechoic points, which are reported mainly in the animals, left–right USLs, rectosigmoid region, bladder, and ureter.

**Laparoscopic evaluation and histology: **The right and left USLs were examined laparoscopically, resection of uterosacral endometriosis was performed, the samples were transferred to the pathology ward, then laparoscopic findings were compared with the results of pathology report. In the absence of visible lesions, a biopsy of the USL was performed. Uterosacral samples were then sent to pathology for histological confirmation. The results of sonography and pathology were compared and analyzed.

**Statistical analysis: **To describe the data, mean, standard deviation, median, amplitude, frequency, and percentage were used. To compare the results of the two methods, chi-square statistical test along with sensitivity, specificity, and positive and negative predictive values were utilized and analyzed using the SPSS 23 statistical software.

## Results

**Patient data: **This study was performed on 80 patients with mean age 34.47±5.94 (mean±SD) years, with the lowest and highest ages being 20 and 50 years, respectively. Mean height, weight, and body mass index (BMI) were 164±5.94 cm, 65.06±1.08 kg, and 24.09±3.91 kg/m^2^ (mean±SD), respectively. Of the total number of patients, 54 (67.5%) were married, and 26 (32.5%) were single (none of the patients were virgins). 

A total of 43 (53.8%) patients were nulliparous, and 37 (46.2%) patients were multiparous. A total of 10 patients from the nulliparous group and 11 patients from the multiparous group had a history of infertility (table 1). A total of 3 (3.8%) patients had no dysmenorrhea, and another 77 (96.2%) subjects complained of menstrual pain. A total of 40 (50%) patients had pelvic pain, whereas an equal number of patients (50%) did not have it. Dyspareunia was found in 45 (56.2% patients), and 35 (43.8%) patients did not have it. The mean severity of menstrual pain, pelvic pain, and dyspareunia was 7.36, 2.67, and 3.76, respectively. Also, the mean duration of dysmenorrhea, pelvic pain, and dyspareunia was evaluated to be 5.03, 1.02, and 1.8 years, respectively. The study found that 37 (46.2%) patients suffered from pain during defecation, 8 (10%) patients complained of dysuria, and 24 (30%) patients complained of pain due to probe pressure during ultrasound. 

Ultrasound and laparoscopic findings, including increased thickness of right and left USLs, the presence of nodules in these ligaments, the presence of ovarian endometrioma, and involvement of the rectum, bladder, and ureter were examined. 

**Figure 1:**
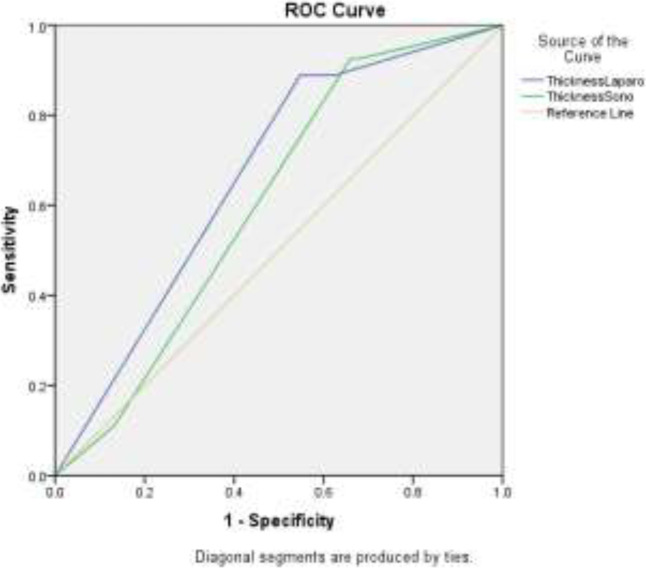
Thickness laparo is a more reliable diagnostic criterion in determining thickness due to p- significance <0.05 compared to ultrasound method and is also a weak biomarker due to the curved surface area of 66%

Regarding the increase in thickness of USLs, as detected in ultrasound, in 60 (75%) patients, it was found in both right and left, in 1 (1.2%) patient, only in the left, and 19 (23.8%) patients did not have this increase in thickness. In contrast, as detected in laparoscopy, the increase in thickness of USLs was found in both the right and the left ligaments in 46 patients (57.5%), 9 (11.2%) patients had this only in the right side, 2 (2.5%) patients had this only in the left side, and it was not found at all 23 (28.8%) patients. Compared to the standard method (laparoscopy histopathology), ultrasound as the gold standard method was found to have sensitivity, specificity, and positive and negative predictive values of 93%, 65%, and 87% and 78.9%, respectively, in detecting increase in USL thickness (table 2) (figure1).

**Table 1:** Demographic data (age, height, weight, parity, and fertility)

**Variable**	**Mean ± SD**	**Minimum**	**Maximum**
Age	34.47±5.94	20	50
Height	164±5.94	152	183
Weight	65.06±1.08	47	94
BMI (kg/m^2^)	24.09±3.91	17.5	35
History of pregnancy	Frequency (100%)	History of infertility	Frequency (100%)
Nulliparous	(53.8%)	Yes (primary)	(12.5%)
		No	(41.25%)
Multiparous	(46.2%)	Yes (secondary)	(13.75%)
		No	(32.5%)

**Table 2:** Increased uterosacral ligament thickness on ultrasound and laparoscopy

**Thickness of the right and left uterosacral ligaments **	**Frequency**		**Percentage**	**Thickness of the right and left uterosacral ligaments **	**Frequency**		**Percentage**
Normal	19		23.8	Normal	23		28.8
Right	0		0	Right	9		11.2
Left	1		1.2	Left	2		2.5
Bilateral	60		75	Bilateral	46		57.5
Total	80		100	Total	80		100
**Sensitivity**		**Specificity**		**Positive predictive value**		**Negative predictive value**	
93%		65%		87%		78.9%	

**Transvaginal sonography findings: **Examination of nodules in the right and left USLs on ultrasound showed that 27 (33.8%) patients had nodules in both right and left, 23 (28.8%) had them only in the right, 15 (18.8%) only in the left, and another 15 (18.8%) did not have them. On laparoscopy, nodules were found in both the right and the left in 30 (37.5%) patients, only 26 (32.5%) patients had them only in the right side, 23 (28.8%) patients only in the left side, and no nodules were found in 1 (1.2%) patient. 

**Figure 2:**
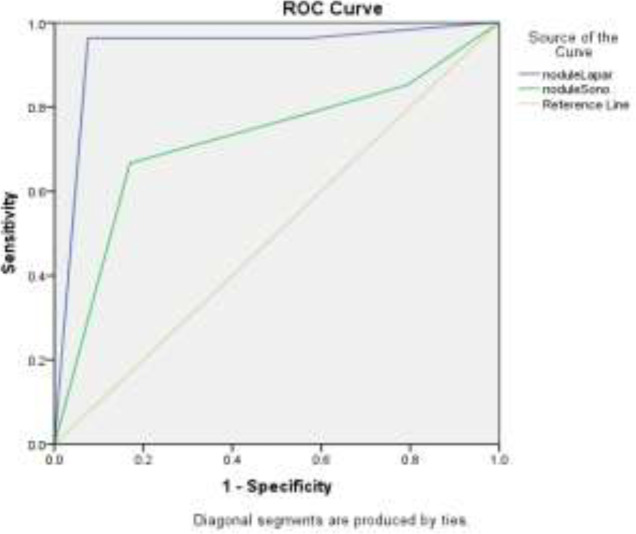
Nodule Lapar and nodule Sono are excellent biomarkers for detecting the number of nodules in patients with 93 and 72% below the rock curve, and laparoscopy is a more accurate diagnostic criterion. Both criteria have a p-value <0.001 and are significant

Compared to the standard method (laparoscopy), ultrasound has sensitivity, specificity, and positive and negative predictive values of 82%, 100%, and 100% and 6.66%, respectively, in the diagnosis of nodules in the USL (table 3) (figure2). Endometrioma in ovaries was reported on ultrasound in 23 (28.7%) patients in both sides, 27 (33.8%) patients had them only in the right, 17 (21.2%) patients only in the left, and 13 (16.2%) did not have them; laparoscopy reported endometrioma in ovaries in 26 (32.5%) patients in both sides, 24 (30%) patients had them only in the right side, 17 (21.2%) patients only in the left side, and 13 cases (16.2%) did not have endometrioma. 

**Figure 3:**
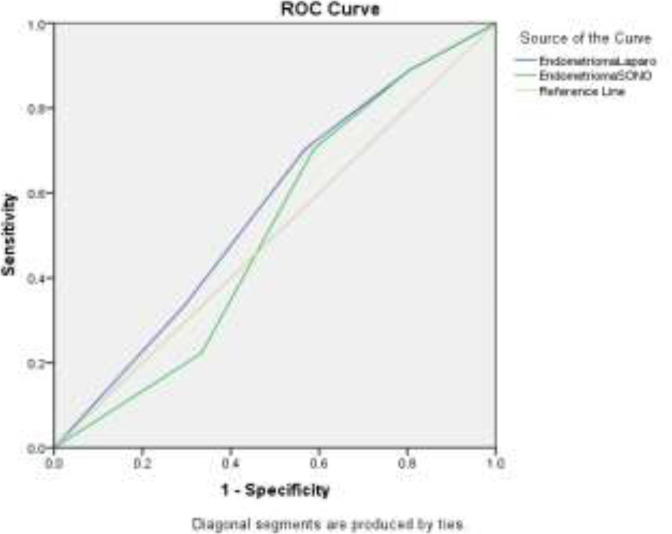
None of them are significant and are not a good diagnostic criterion for endometriosis

Compared to laparoscopy, ultrasound as the gold standard method has sensitivity, specificity, and positive and negative predictive values of all 100% in the diagnosis of endometrioma (table 4) (figure 3).

 With the involvement of the rectum, bladder, and ureter, on ultrasound, rectum involvement was reported in 31 (38.8%) patients, bladder involvement in 3 (3.8%) patients, simultaneous rectum and bladder involvement in 3 (3.8%) patients, simultaneous rectum and ureter involvement in 1 (1.2%) patient, and no involvement was found in 42 (52.5%) patients.

Laparoscopic findings: On laparoscopy, rectal involvement was found in 27 (33.8%) patients, bladder involvement in 5 (6.2%) patients, both rectal and bladder involvement was found in 16 (20%) patients, both rectal and ureteral involvement in 3 (3.8%) patients, all rectal, bladder and ureter involvement in 1 (1.2%) patient, and 28 (35%) patients were negative for this variable, that is, no involvement found. Compared to laparoscopy, ultrasound as the gold standard method has sensitivity, specificity, and positive and negative predictive values of 71%, 96.4%, and 97.3%, and 64.2%, respectively, in the diagnosis of visceral involvement (rectum, urinary bladder, and ureter).

**Table 3:** Nodules in uterosacral ligament on ultrasound and laparoscopy

**Nodule in sonography**	**Frequency**		**Percentage**	**Nodule in laparoscopy**	**Frequency**		**Percentage**
Normal	15		18.8	Normal	1		1.2
Right	23		28.6	Right	26		32.5
Left	15		18.8	Left	23		28.8
Bilateral	27		33.8	Bilateral	30		37.5
Total	80		100	Total	80		100
Sensitivity		Specificity		Positive predictive value		Negative predictive value	
82%		100%		100%		6.66%	

**Table 4:** Endometrioma in ovaries via ultrasound and laparoscopy

**Endometrioma in the right and left ovaries in sonography**	**Frequency**		**Percentage**	**Endometrioma in the right and left ovaries in laparoscopy**	**Frequency**		**Percentage**
Normal	13		16.2	Normal	13		16.2
Right	27		33.8	Right	24		29.8
Left	17		21.25	Left	17		21.25
Bilateral	23		28.75	Bilateral	26		32.75
Total	80		100	Total	80		100
**Sensitivity**		**Specificity**		**Positive predictive value**		**Negative predictive value**	
100%		100%		100%		100%	

## Discussion

In our study, the diagnostic sensitivity, specificity, positive and negative predictive values of TVS were compared with laparoscopy (confirmed with pathology). In the diagnosis of increase in USL thickness, the diagnostic sensitivity, specificity, positive and negative predictive values were 93%, 65%, and 87%, and 78.9%, respectively. Zhou et al. declared that the sensitivity, specificity, positive and negative predictive values for USL sonography diagnosis were 65%, 92%, 7.8% and 0.38%, respectively (22). They also reported that TVS provides an excellent comprehensive diagnostic performance for DIE which is similar to our result. The TVS and laparoscopic data by Pattanasri et al. in 2020 revealed that the specificity and sensitivity in the diagnosis of deep infiltrative endometriosis (DIE) were 46.1% and 83.3%. Also, the sensitivity of TVS in diagnosis of uterosacral ligament DIE was 70.9% (23). Similar to our study, Alborzi et al. achieved sensitivity, specificity, and positive and negative predictive values of 83.3%, 46.1%, and 85.7% and 41.6%, respectively, for DIE (24).

The diagnostic sensitivity, specificity, positive and negative predictive values of uterosacral endometriosis in our study were 82%, 100%, 100% and 6.66%, respectively. Hudelist et al. have stated that TVS has a sensitivity of 40% and a specificity of 95.6% in the diagnosis of uterosacral endometriosis (25). Probable reasons for finding lower sensitivity compared to our results may be the lower skill set of the operator, use of transrectal sonography in some patients, and less severity of lesions. Zhang et al. found sensitivity, specificity, and positive and negative predictive values of 90.9%, 96.4% and 88.2% and 94%, respectively, of TVS in the diagnosis of USL endometriosis (19). Nisenblat et al. in 2016 concluded that the laparoscopic diagnostic of pelvic endometriosis as a golden standard is more accurate than TVS, clinical data and serum biomarkers alone (26). However, Ghatresamani et al. evaluated transvaginal–transrectal sonography in DIE, which showed a diagnostic sensitivity of 100% in pelvic masses, and sensitivity and specificity of 50% and 100%, respectively, in the diagnosis of bladder involvement (20). Also, Holland et al. reported that TVS has a high specificity in the diagnosis of endometriosis and false-positive results are rare (21). 

The lower sensitivity may be due to the lower skill set of the operator, smaller size, location and number of lesions, the use of a lower model of ultrasonography or not using combination of diagnostic techniques with each other. Tammaa et al. in 2015 discovered that using a trained specialist dramatically increases the accuracy and reproducibility of TVS for diagnosing DIE (27). Saba et al. in 2012 indicated that combination of TVS with MRI increase the accuracy of rectosigmoid endometriosis diagnosis up to 95% (28). All results in these reports in accordance to the findings in our study that the diagnostic accuracy of sonography depends on the location and number of lesions.

Our data revealed that the diagnosis value of all four parameters for ovarian endometrioma were 100%. Vesical endometriosis was examined by TVS in a study by Savelli et al., where they reported sensitivity, specificity, and positive and negative predictive values of 44%, 100%, and 100% and 95%, respectively (29). Again, the lower sensitivity may be a result of the lower skill set of the operator, or smaller and less severe lesions. Yazbeck et al. reported sensitivity and specificity of 44% and 98% of TVS in the evaluation of severe pelvic adhesions (30). However, Ayachi et al., in 2017 estimate the sensitivity (96.3%) and specificity (92.6%,) of transvaginal sonographic (TVS) in women with previous abdominopelvic surgery (31). 

Their results also showed that there is a close relationship between adhesion and TVS sliding sign. The ultrasound method in the diagnosis of visceral involvement has sensitivity, specificity, positive and negative predictive values of 71%, 96.4%, 97.3%, and 64.2%, respectively. In a study by Saccardi et al., sensitivity and specificity of TVS in USL assessment were found to be 88.9% and 95.6%, respectively, which is similar to the findings in our study (32). Abrao et al. reported the sensitivity and specificity of TVS in the diagnosis of retro-cervical endometriosis 95% and 98%, respectively. They reported the positive predictive value at 98% (33). They also found the sensitivity, specificity, and positive predictive value of TVS in the evaluation of rectosigmoid involvement at 98%, 100%, and 100%, respectively. These higher values compared to our study may be due to the better skill set of the operator, more severity of involvement, etc. Goncalves et al. reported 81% sensitivity and 99% specificity of TVS in the evaluation of rectosigmoid endometriosis (34). On the other hand, Ferrero et al. reported that the accuracy of TVS in diagnosing the presence of rectosigmoid endometriosis was 92.3% (35).

**In conclusion we conclude that TVS has an acceptable sensitivity in the diagnosis of uterosacral involvement: 93% in evaluating thickening and 82.5%** **i** **n the evaluation of hypo-echo nodules; however, specificity in these two cases was 65% and 100%, respectively. Despite acceptable sensitivity and specificity in the diagnosis of adnexal endometriomas, and urinary bladder and rectal involvement, involvement cannot be ruled out in the presence of negative results with a sensitivity of 71%.**
